# Discontinuation versus continuation of renin–angiotensin system inhibitors in chronic kidney disease stage 3–5 patients: a systematic review and meta-analysis

**DOI:** 10.3389/fphar.2025.1646969

**Published:** 2025-09-26

**Authors:** I-Wen Chen, Yi-Hsuan Lin, Vin-Cent Wu, Jui-Yi Chen, Ming-Hsien Wu

**Affiliations:** ^1^ Division of Endocrinology and Metabolism, Department of Internal Medicine, Chang Gung Memorial Hospital, Taoyuan, Taiwan; ^2^ College of Medicine, Chang Gung University, Taoyuan, Taiwan; ^3^ Department of Internal Medicine, National Taiwan University Hospital, Taipei, Taiwan; ^4^ Division of Nephrology, Department of Internal Medicine, Chi Mei Medical Center, Tainan, Taiwan; ^5^ Department of Health and Nutrition, Chia Nan University of Pharmacy and Science, Tainan, Taiwan; ^6^ Division of Endocrinology and Metabolism, Department of Internal Medicine, New Taipei Municipal TuCheng Hospital (built and operated by Chang Gung Medical Foundation), New Taipei City, Taiwan

**Keywords:** angiotensin-converting enzyme inhibitors, angiotensin II receptor blockers, chronic kidney disease, continuation, discontinuation

## Abstract

**Background:**

Renin-angiotensin system inhibitors (RASi), comprising angiotensin-converting enzyme inhibitors (ACEi) or angiotensin II receptor blockers (ARB) are known for cardio- and renoprotection. However, there is uncertainty regarding the continuation of ACEi or ARB treatment in patients with chronic kidney disease (CKD) stages 3–5.

**Methods:**

In this meta-analysis, we systematically searched all relevant studies published in PubMed, Embase, and the Cochrane Library up to 30 May 2024. Our objective was to assess the impacts of continuation or discontinuation of RASi in patients with CKD stages 3–5 on all-cause mortality, end-stage kidney disease, major adverse cardiovascular events (MACE), and hyperkalemia. We rated the certainty of the evidence using the Cochrane methods and the GRADE approach.

**Results:**

The search identified 520 studies, of which 8 studies, encompassing a total of 243,775 patients, were included in the analysis. The incidence of all-cause mortality was 40.3% (29,993 out of 74,447 patients), while ESKD occurred in 27.9% (8,992 out of 32,191 patients), MACE in 37.3% (11,225 out of 30,059 patients), and hyperkalemia in 39.4% (8,533 out of 21,642 patients). Pooled analysis revealed that patients who discontinued RASi therapy had a higher risk of developing ESKD compared to those who continued treatment [Hazard ratio (HR): 1.40, 95% confidence interval (CI): 1.19–1.65, P < 0.001], but a lower risk of hyperkalemia [Odds ratio (OR): 0.68, 95% CI: 0.60–0.77, P < 0.001]. There were no significant differences between the groups in all-cause mortality (HR: 1.34, 95% CI: 0.91–1.95, P = 0.135) and MACE (OR: 1.27, 95% CI: 0.93–1.73, P = 0.138).

**Conclusion:**

Patients who discontinued RASi therapy exhibited a higher risk of developing ESKD but a reduced risk of hyperkalemia compared to those who continued RASi treatment. However, there were no significant differences in all-cause mortality and MACE between the two groups.

**Systematic Review Registration:**

identifer, PROSPERO (CRD42023494698).

## Introduction

The renin-angiotensin system (RAS) promotes inflammation and fibrosis, enhances sympathetic nervous system activity, increases sodium and chloride reabsorption in the tubules, elevates aldosterone release, induces arteriolar constriction, and stimulates anti-diuretic hormone (ADH) secretion (Sawaf et al., 2022). Angiotensin-converting enzyme inhibitors (ACEi) and angiotensin II receptor blockers (ARB) can mitigate these effects to reduce myocyte hypertrophy, arrhythmogenic effects, and fibrosis in both the heart and kidneys (Mukoyama and Kuwabara, 2022).

For chronic kidney disease (CKD) patients, the use of ACEi/ARB is associated with a lower risk of major cardiovascular events (Xie et al., 2016) and mortality (Wright et al., 2002). Additionally, ACEi/ARB users have a reduced risk of doubling of serum creatinine levels (Wright et al., 2002), and a decreased incidence of end-stage kidney disease (ESKD) (Deng et al., 2022). Therefore, the Kidney Disease: Improving Global Outcomes (KDIGO) guidelines suggests the substantial benefits of ACEi and ARB in managing CKD. These guidelines strongly advocate for the use of ACEi or ARB in individuals with CKD (Kidney Disease: Improving Global Outcomes Blood Pressure Work, 2021; Kidney Disease: Improving Global Outcomes Diabetes Work, 2022).

However, the administration of RAS inhibitors (RASi) may lead to a temporary decrease in estimated glomerular filtration rate (eGFR). This effect is attributed to the reduction in systemic blood pressure and the vasodilatory impact on efferent arterioles, leading to a consequent decrease in intraglomerular pressure (Burnier, 2020). Besides, the use of RASi is associated with an increased risk of hyperkalemia, primarily due to their role in inhibiting aldosterone secretion, which in turn impairs the kidneys’ ability to excrete potassium. This risk is particularly elevated in patients with deteriorating renal function (Raebel, 2012; Bandak et al., 2017).

Multiple clinical trials have demonstrated that blockade of the renin–angiotensin system is reno-protective and effectively reduces CKD progression. However, most trials excluded participants with advanced CKD, especially stage 4 and 5 (Weir et al., 2018). Therefore there is no definitive conclusion on the impact of continued RASi use on kidney function and the risk of hyperkalemia in these patients. We undertook a meta-analysis to evaluate the impact of discontinuing versus continuing RASi on clinical outcomes among patients with CKD stage 3–5.

## Methods

### Methodology

This meta-analysis adhered to the principles outlined in the Preferred Reporting Items for Systematic Reviews and Meta-Analyses (PRISMA) statement (Higgins et al., 2011) (Supplementary Material 1).

### Search strategy

Three investigators (I.W. Chen, Y.H. Lin, and M.H. Wu) conducted electronic database searches in PubMed, EMBASE, and the Cochrane Library for relevant studies published from the inception of 30 May 2024. We utilized the subsequent sets of keywords and their combinations, (1) “chronic kidney disease” and (2) “Renin angiotensin system inhibitor” or “Angiotensin converting enzyme inhibitor” or “Angiotensin receptor antagonist” and (3) “Discontinue” or “continue” (Supplementary Material 2).

We also used Medical Subject Headings (MeSH) terms to improve the search’s sensitivity and identify additional pertinent studies. Additionally, we conducted a manual review of the reference lists of the included articles to identify potentially suitable studies. After eliminating duplicate entries, the three authors (I.W. Chen, Y.H. Lin, M.H. Wu) individually carried out an initial assessment of the studies by evaluating their titles and abstracts. Subsequently, they conducted a comprehensive examination of eligible studies by reviewing their complete texts. Discrepancies that arose during the search and study selection process were resolved through discussion. We submitted the protocol for our systematic review to PROSPERO for prospective registration (CRD42023494698).

### Eligibility criteria

The inclusion criteria were as follows: (a) individuals aged 18 years or older; (b) CKD stage 3–5 defined by a GFR less than 60 mL/min per 1.73 m^2^; (c) the administration of RASi, including either ACEi or ARB; (d) the reporting at least one of the following outcomes, such as all-cause mortality, ESKD and cardiovascular events. Exclusion criteria included: (a) Studies including animal or healthy human subjects; (b) Studies including pregnant or lactating patients; (c) Comparison with RASi with other anti-hypertensive drugs; (d) case reports, editorials, and reviews; (e) no control group for comparing the effects of continuing versus discontinuing ACEi or ARB therapy were all excluded.

### Data extraction and quality assessment of the included studies

The characteristics of these studies encompassed various details, including the first author’s name, year of publication, study design, data source, study groups, sample size, duration of follow-up, and the stage of CKD. The outcomes evaluated in this meta-analysis encompassed all-cause mortality (Qiao et al., 2020; Walther et al., 2021; Bhandari et al., 2022; Yang et al., 2023; Leon et al., 2022) as well as ESKD (Qiao et al., 2020; Walther et al., 2021; Bhandari et al., 2022; Nakayama et al., 2022; Yang et al., 2023; Leon et al., 2022), major adverse cardiovascular events (MACE) (Qiao et al., 2020; Fu et al., 2021a; Yang et al., 2023) and hyperkalemia (Hou et al., 2006; Qiao et al., 2020; Yang et al., 2023). ESKD was defined as initiation of dialysis or kidney transplantation. Cardiovascular events were defined as non-fatal stroke, non-fatal myocardial infarction, heart failure, percutaneous coronary intervention, or coronary artery bypass and cardiovascular death. Serum potassium level greater than 5.5 mEq/L was ascertained as hyperkalemia. The definitions of MACE and hyperkalemia varied across included studies and are summarized in Supplementary Material 5.

We conducted subgroup analyses based on randomized controlled trials (RCTs) versus non-RCTs. To assess the quality of the studies, we employed the Newcastle-Ottawa Scale (NOS) for cohort studies and Version 2.0 of the Cochrane risk of bias tool for randomized trials (RoB 2.0) in the case of RCTs. In this meta-analysis, the GRADE (Grading of Recommendations, Assessment, Development and Evaluations) system was used to assess the quality of evidence and the strength of recommendations (Supplementary Material 6).

### Statistical analysis

To determine the magnitude of the effect for outcomes in this meta-analysis, we employed a hazard ratio (HR) in conjunction with a 95% confidence interval (CI). For our meta-analysis of outcomes, we applied random effects methods, utilizing the DerSimonian-Laird estimator for variance. This approach was chosen to compute the combined effect size for each outcome, considering the recognized clinical and methodological diversity among the studies (Veroniki et al., 2016). All statistical analyses were conducted using Comprehensive Meta-Analysis (Version 3.3.070, dated 20 November 2014).

### Trial sequential analysis

Trial sequential analysis (TSA) in this meta-analysis was applied to minimize the risk of false-positive or false-negative results (Brok et al., 2008; Kang, 2021). This method determined the adequacy of evidence when the cumulative Z-curve either crossed the trial sequential monitoring boundary or reached the futility area, eliminating the need for further studies. If the Z-curve did not achieve these thresholds and the required information size (RIS) was not fulfilled, it suggested that the current evidence was insufficient, calling for additional research for verification (Liu et al., 2016). The RIS in our TSA was based on a projected 10% reduction in relative risk (RR). We maintained the type I error (α) at 0.05 (two-sided) and employed a power (1-β) of 0.90 to calculate the RIS. The proportion of control events was calculated using data from the comparator group (Liu et al., 2019). The TSA was executed using TSA software Version 0.9.5.10 Beta.

## Results

### Study search outcomes and included patients

**FIGURE 1:**
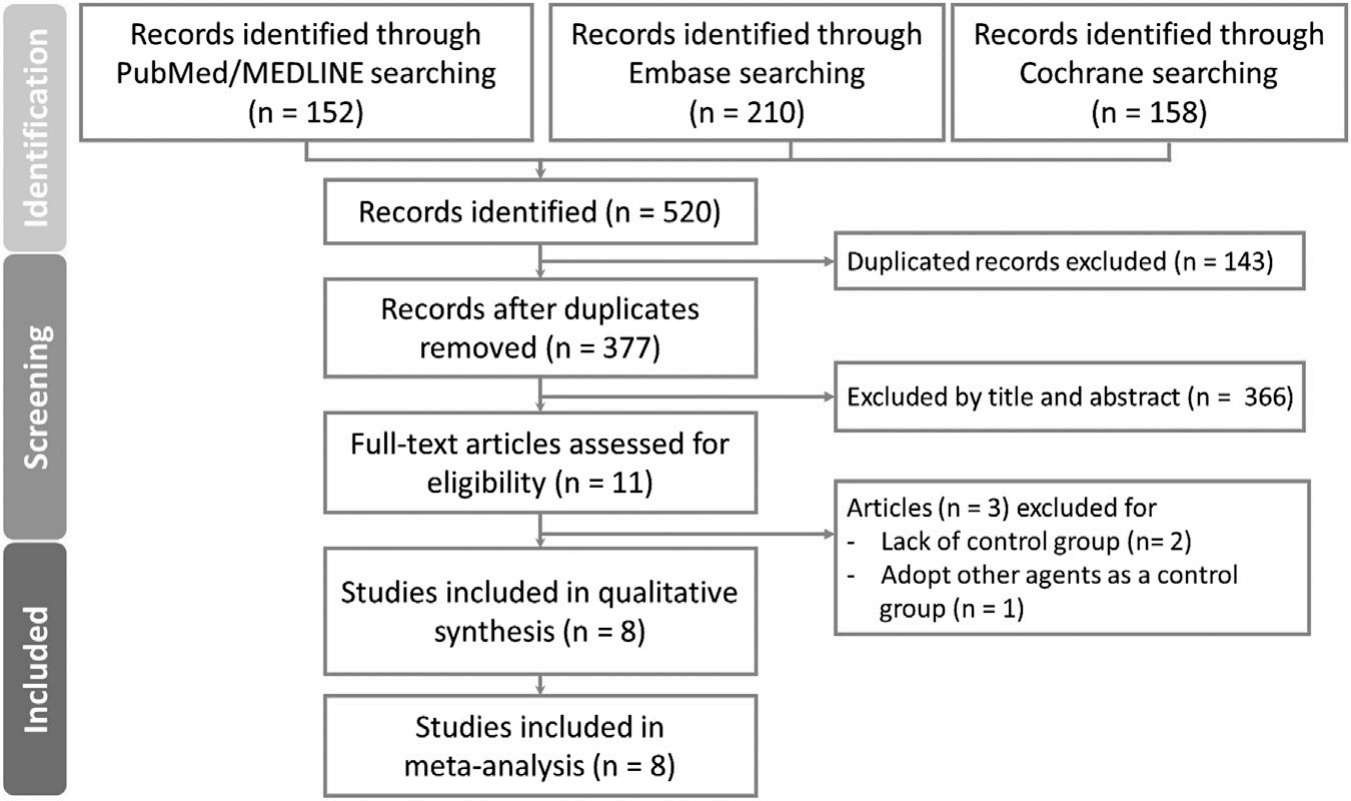
PRISMA flow diagram for systematic reviews which included searches of databases and registers.

As shown in Figure 1, a total of 520 articles were identified from PubMed, Embase, and Cochrane databases, respectively. Out of these, 143 articles were excluded due to duplication. Subsequently, 377 articles underwent screened based on their titles and abstracts. Following this initial screening, 11 articles were evaluated for full eligibility, leading to the exclusion of three articles (two lacked control group (Ahmed et al., 2010; Leon, 2020), and one used other agents as a control group (Fu et al., 2021a)). Finally, eight articles, including 243,775 patients with complete data and outcomes of interest, were enrolled for the final meta-analysis (Figure 1).

Five studies were retrospective studies (Qiao et al., 2020; Walther et al., 2021; Fu et al., 2021b; Nakayama et al., 2022; Leon et al., 2022), while one was a prospective cohort study (Yang et al., 2023). The remaining two studies were randomized control studies (Bhandari et al., 2022; Hou et al., 2006). Baseline characteristics and outcomes for the included studies are presented on Tables 1 and 2, respectively.

**TABLE 1:** Baseline characteristics of the included studies.

Author name (year)	Data source	Study design	Comparison (n)	Sample size (n)	Age (years)	Female (%)	CKD stage	Mean/Median GFR (mL/min/1.73 m^2^)	HTN (%)	DM (%)	CAD (%)	CHF (%)	Stroke (%)
Hou et al. (2006)	Nanfang Hospital Renal Division	RCT	C:112 D: 112	224	44.8	49	sCr 3.1–5.0	ACEi: 26.3 ± 5.3^a^ Placebo: 25.8 ± 5.3^a^	90.5	NR	NR	2.7	NR
Qiao et al. (2020)	Geisinger Health System	Retrospective, propensity score–matched cohort study	C:1,205 D:1,205	2,410	73.7 ± 12.6	61.6	Stage 4–5	C: 23.9 ± 5.3^a^ D: 23.6 ± 5.3^a^	NR	48.2	44.0	32.9	20.0
Fu et al. (2021)	Swedish Renal Registry	Retrospective, observational study	C: 8,701 D: 1,553	10,254	72 (IQR, 62–79)	35.7	Stage 4–5	23 (IQR, 18–27)	88.7	49.5	21.6	28.0	15.8
Walther et al. (2021)	Veterans Affairs healthcare system	Retrospective cohort study	C: 5,896 D: 135,356	141,252	73.7 ± 10.4	3.0	Stage 3–4	49.3 ± 13.8^a^	NR	42.5	11.6	17.4	21.2
Bhandari S et al. (2022)	39 centers in the United Kingdom.	RCT, multicenter, open-label trial, randomize	C: 205 D: 206	411	62.1 ± 13.1	31.6	Stage 4–5	C: 13.3 ± 0.6^a^ D: 12.6 ± 0.7^a^	72.0	37.2	8.8	4.0	6.0
Nakayama et al. (2022)	Keio University School of Medicine Ethics Committee	Retrospective cohort study	C: 136 D: 51 Non user: 144 New user: 3	334	70 (IQR, 59–79)	28.4	Stage 5	9.1 (IQR, 7.3–11.2)	94.9	44.0	17.7	28.2	18.6
Leon et al. (2022)	The Manitoba Centre for Health Policy	Retrospective cohort study	C: 4,674 D: 2,526	7,200	72.4 ± 13.4	47.5	Stage 3–5	40.9 ± 13.8^a^	100	63.1	NR	37.2	14.9
	International Credential Evaluation Service (ICES)	Retrospective cohort study	C: 61,308 D: 9,982	71,290	79.5 ± 7.5	51.7	Stage 3–5	41.2 ± 12.6^a^	88.9	58	NR	21.8	6.8
Yang A et al. (2023)	Hong Kong-Diabetes-Surveillance-Database	Prospective cohort study	C: 8,634 D: 1,766	10,400	73.1 ± 11.4	53.5	Stage 4–5	C: 29.0 ± 11.0^a^ D: 29.9 ± 13.4^a^	NR	100	10.1	9.4	6.5

Data are presented as number (percentage) of patients and as either mean ± standard deviation (SD) or median (interquartile range, IQR), unless specified otherwise.

^a^ Mean ± SD.

C, continue; D, discontinue; CAD, coronary artery disease; CHF, congestive heart failure; DM, diabetes mellitus; eGFR, estimated glomerular filtration rate (mL/min/1.73 m^2^); HTN, hypertension; IQR, interquartile range; NR, not reported; RASi, renin - angiotensin system inhibitor; RCT, randomized controlled trial; sCr, serum creatinine.

**TABLE 2:** Summary of included comparative studies for outcomes evaluatio**n**.

Author name (year)	Intervention	Duration	Follow up duration	Mortality (%) (Continue vs. discontinue)	ESKD (%) (Continue vs. discontinue)	Primary outcome	Secondary outcome
Hou et al. (2006)	Benazepril	1999/05–2001/05	3.4 years (Range, 2–5 years)	0.9 vs. 0.0	NR	Doubling of the serum creatinine level, ESKD, or Death.	Changes in the level of proteinuria and the rate of progression of renal disease.
Qiao et al. (2020)	ACEi/ARB	2004/01/01–2018/12/31	Median 2.9 years (IQR, 1.3–5.0 years)	29.4 vs. 35.1	6.6 vs. 7.0	All-cause mortality	MACE and ESKD
Fu et al. (2021)	RASi	2007–2017	5 years	54.4 vs. 40.8	36.1 vs. 27.9	All-cause mortality	MACE and KRT (defined as undergoing kidney transplantation or initiating maintenance dialysis).
Walther et al. (2021)	ACEi/ARB	2005/01/01–2015/12/31	4.47 years (IQR, 2.20–7.31)	NR	NR	Death and ESKD	
Bhandari S et al. (2022)	RASi	2014/7/11–2018/7/19	Median 3 years (2.7 ± 0.8 years)	10.7 vs. 9.7	56.1 vs. 62.1	initiation of renal-replacement therapy (dialysis or transplantation)	The development of ESKD; a composite of a decrease of more than 50% in the eGFR or the initiation of RRT; hospitalization; blood pressure; exercise capacity; and quality of life.
Nakayama et al. (2022)	RASi	2014/04–2021/03	6 months	NR	NR	Unexplained dialysis	
Leon et al. (2022)	RASi	2007/1/1–2017/03/31	>9 years	NR	NR	All-cause mortality	CV mortality, fatal and non-fatal CV events, dialysis initiation
	RASi	2007/1/1–2017/03/31	>9 years	NR	NR	All-cause mortality	CV mortality, fatal and non-fatal CV events, dialysis initiation
Yang et al. (2023)	RASi	2002–2019	Median 3.6 years (IQR, 2.1–5.8)	34.8 vs. 40.3	28.1 vs. 25.1	Death, MACE, HF, ESKD, and all-cause mortality.	

ACEi, angiotensin converting enzyme inhibitors; ARB, angiotensin receptor blockers; CABG, coronary artery bypass graft; CV, cardiovascular; ESKD, end-stage kidney disease; eGFR, estimated glomerular filtration rate (mL/min/1.73 m^2^); HF, heart failure; KRT, kidney replacement therapy; MACE, major adverse cardiovascular events; MI, myocardial infarction; NR, not reported; PCI, percutaneous coronary intervention; RASi, renin-angiotensin system inhibitor; RRT, renal replacement therapy; sCr, serum creatinine.

The follow-up durations in the eight articles ranged from 6 months to up to 9 years. Moreover, the range of mean/median baseline eGFR was 9.1–49.3 mL/min/1.73 m^2^. Among the included articles, the primary outcome was defined as patient mortality in six articles (Hou et al., 2006; Qiao et al., 2020; Fu et al., 2021b; Walther et al., 2021; Leon et al., 2022; Yang et al., 2023), while ESKD served as the primary outcome in three articles (Hou et al., 2006; Walther et al., 2021; Yang et al., 2023). ESKD was defined as secondary outcomes in another article (Bhandari et al., 2022). One article focused on the incidence of unplanned dialysis initiation as its primary outcome (Nakayama et al., 2022).

### Quality of enrolled trials

The studies spanned various years (2006–2022) and exhibited substantial variation in sample sizes from 104 to 141,252 patients. Besides, the data were collected from different sources by the authors, including population databases, health insurance systems, and/or multiple hospitals. Patients from all enrolled studies were categorized into either the discontinued-RASi and continued-RASi groups, and outcomes were compared between the two groups (Tables 1 and 2).

The results of quality assessment, based on the NOS for included the six cohort studies (Qiao et al., 2020; Fu et al., 2021b; Walther et al., 2021; Nakayama et al., 2022; Yang et al., 2023; Leon et al., 2022), ranged from 7 to 8 (Supplementary Table S1A). This indicated that six studies demonstrated good methodological quality. In the studies of Qiao et al., Nakayama et al., Yang A et al., and Silva J. Leon et al., there were patients who expired or progressed to ESKD at the beginning of follow-up period (Qiao et al., 2020; Nakayama et al., 2022; Yang et al., 2023; Leon et al., 2022). Besides, the study conducted by Walther et al., no additional confounders was adjusted in the analysis (Walther et al., 2021). Therefore, four of six cohort studies scored 7 points by NOS. The RoB 2.0 was adopted for two randomized control studies. Low risk was assessed in the study of Bhandari et al. (2022). However, moderate risk was assessed in Hou et al., which was unable to discern differences in each component of the kidney composite outcome (Hou et al., 2006). Thus some concerns were judged in the domain “Bias in selection of the reported result” (Supplementary Table S1B).

### All-cause mortality

**FIGURE 2:**
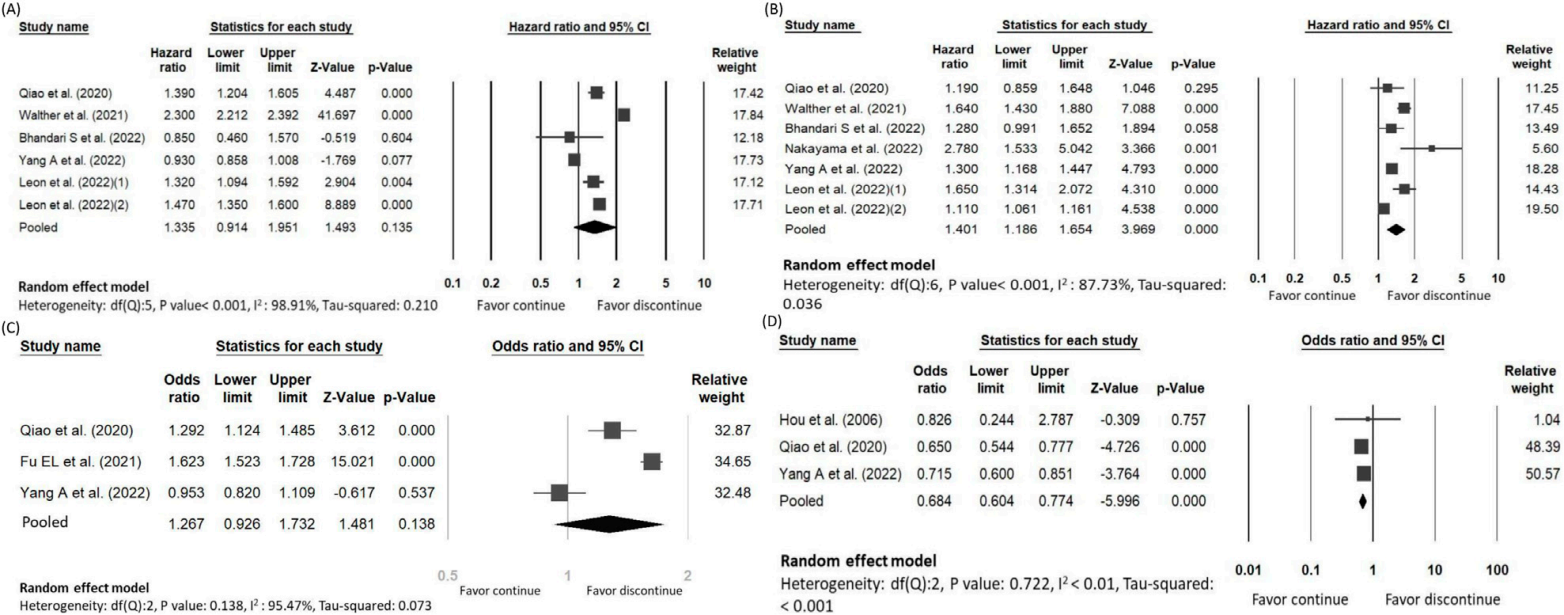
Forest plots showing the pooled risk of **(A)** all-cause mortality, **(B)** ESKD, **(C)** MACE and **(D)** hyperkalemia between continuing and discontinuing ACEi/ARB groups.

The main outcome of interest assessed in five studies encompassing 29,993 patients and 12,096 deaths with overall all-cause mortality rate of 40.3% (Qiao et al., 2020; Fu et al., 2021b; Bhandari et al., 2022; Yang et al., 2023; Hou et al., 2006; Leon et al., 2022). The risk of all-cause mortality did not demonstrate statistical difference between the discontinued-RASi users and continued-RASi users [OR: 0.94, 95% CI: 0.56–1.58, P = 0.816, certainty of evidence (COE): moderate] (Supplementary Figure S1A) and the funnel plot showed symmetrical distributions (Supplementary Figure S2A). Additionally, HR for mortality was reported in five articles (Qiao et al., 2020; Walther et al., 2021; Bhandari et al., 2022; Yang et al., 2023; Leon et al., 2022) and no statistically significant difference was observed between discontinued-RASi users and continued-RASi users (HR: 1.34, 95% CI: 0.91–1.95, P = 0.135, I^2^ = 98.91%) with considerable heterogeneity among the study results (I^2^ = 98.91%) (Figure 2A).

### The risk of ESKD

**FIGURE 3:**
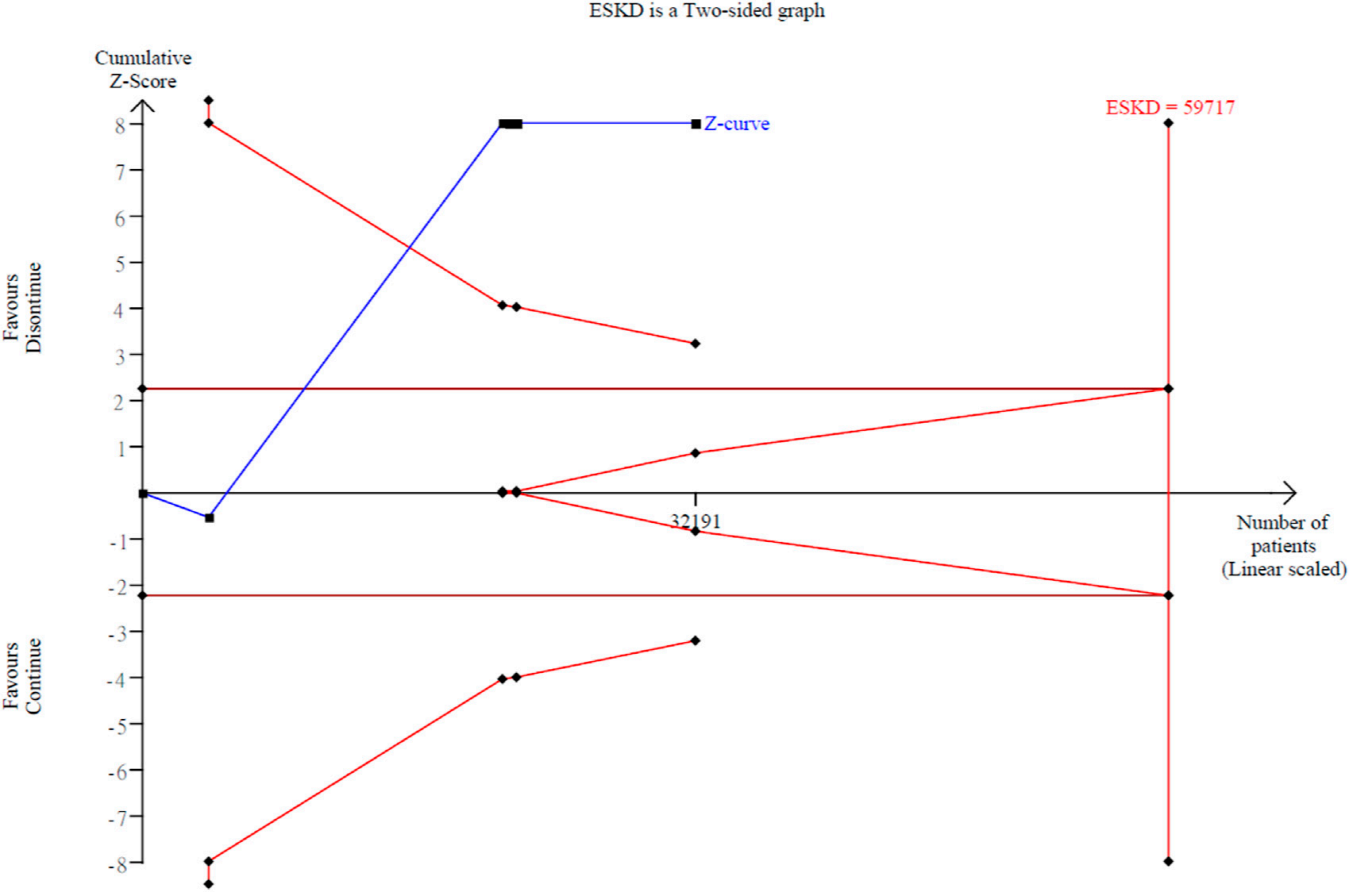
Trial sequential analysis over the ESKD events with continuing versus discontinuing of ACEi/ARB groups.

Our secondary outcome of interest was the occurrence of ESKD in 8,992 out of 32,191 patients (27.9%) across the six studies, with a mean follow-up of 2.92 years (Qiao et al., 2020; Bhandari et al., 2022; Fu et al., 2021b; Yang et al., 2023; Nakayama et al., 2022). There was no statistical significance about the difference of ESKD between the discontinued-RASi users and continued-RASi users (OR: 1.05, 95% CI: 0.80 to 1.39, P = 0.708, COE: very low) (Supplementary Figure S1B) and the funnel plot showed symmetrical distributions (Supplementary Figure S2B). In six articles reporting HR for ESKD (Qiao et al., 2020; Walther et al., 2021; Bhandari et al., 2022; Yang et al., 2023; Nakayama et al., 2022; Leon et al., 2022), pooled results indicated a higher risk of ESKD in patients in the discontinued-RASi users compared to the continued-RASi users (HR: 1.40, 95% CI: 1.19–1.65, P < 0.001, I^2^ = 87.73%) (Figure 2B). For the incidence of ESKD, the TSA indicated the accrued information size was 59,717. The cumulative *Z*-curve crossed the conventional boundary and even the monitoring boundary, reached the superiority zone, indicating that discontinued-RASi users has higher risk of ESKD than the continued-RASi users (Figure 3). However, the cumulative z-curve did not reach the line of required information size.

### The risk of MACE

11,225 out of 30,059 patients had MACE (37.3%) according to three studies (Qiao et al., 2020; Fu et al., 2021b; Yang et al., 2023). The pooled findings indicated that discontinued-RASi users had a higher risk of developing MACE compared to continued-RASi users. However, this difference did not reach statistical significance (OR: 1.27, 95% CI: 0.93–1.73, P = 0.138, I^2^ = 95.47%, COE: very low) (Figure 2C).

### The risk of hyperkalemia

We evaluated risk of hyperkalemia based on three included studies, involving 8,533 patients (Hou et al., 2006; Qiao et al., 2020; Yang et al., 2023). Among them, 3,362 individuals experienced hyperkalemia (39.40%). The risk of hyperkalemia was lower in patients in the discontinued-RASi users compared to the continued-RASi users (OR: 0.68, 95% CI: 0.60–0.77, P < 0.001, I^2^ < 1.00%, COE: low), as depicted in Figure 2D.

### Subgroup analysis of RCTs versus non-RCTs

We conducted a subgroup analysis according to whether RCTs or non-RCTs in our included studies. For non-RCT trials, the pooled risk of all-cause mortality revealed no statistical significance between the two groups (HR: 1.42, 95% CI: 0.95–2.13, P = 0.088) (Supplementary Figure S3A). In addition, the pooled risk of ESKD was higher in discontinued-RASi users compared to the continued-RASi users (HR: 1.42, 95% CI: 1.18–1.72, P < 0.001) (Supplementary Figure S3B). The risk of hyperkalemia was lower in the discontinued-RASi users than the continued-RASi users (OR: 0.68, 95% CI: 0.60–0.77, P < 0.001) (Supplementary Figure S3C).

### Subgroup analysis of the CKD stage

We conducted a subgroup analysis according to the CKD stage of patients in our included studies. The pooled risk of all-cause mortality revealed no statistical significance between the two groups whether CKD stage 3 or 4 (HR: 1.65, 95% CI: 0.79–3.42 in CKD stage 3; HR: 1.08, 95% CI: 0.57–2.05 in CKD stage 4) (Supplementary Figure S4A). However, the pooled risk of ESKD was higher in discontinued-RASi users compared to the continued-RASi users in CKD stage 4 (HR: 1.31, 95% CI: 1.06–1.62) but no significant difference in CKD stage 3 (HR: 1.43, 95% CI: 0.81–2.54) (Supplementary Figure S4B).

## Discussion

This study incorporated trials encompassing patients with CKD stages 3–5 to evaluate the differential impacts of discontinuing versus continuing RASi therapy. Patients who discontinued RASi exhibited a higher risk of progression to ESKD compared to those who maintained the therapy. However, no significant difference in mortality risk was observed between the two cohorts. Furthermore, the discontinuation of RASi was associated with a reduced incidence of hyperkalemia in contrast to the continued use of RASi.

Across the included studies, definitions of MACE and hyperkalemia were heterogeneous. Some cohorts adopted conventional 3-point MACE (cardiovascular death, nonfatal myocardial infarction, and nonfatal stroke), whereas others included additional endpoints such as revascularization, arrhythmia, or even all-cause mortality as part of the composite. Similarly, thresholds for hyperkalemia ranged from ≥5.5 to ≥6.0 mmol/L, with some studies relying on registry-based ascertainment and others reporting only trial-adjudicated events. Such variability may alter absolute event rates and bias comparative estimates, thereby limiting the interpretability of pooled results. In recognition of this limitation, we refrained from cross-study pooling of MACE and hyperkalemia, instead reporting them descriptively, and emphasized endpoints with consistent definitions (all-cause mortality and ESKD).

In interpreting observational evidence, it is important to note that the Geisinger Health System analysis (Qiao et al., 2020) was not a randomized trial but a propensity score–matched cohort study comparing patients who discontinued ACEi/ARB after eGFR fell <30 mL/min/1.73 m^2^ with matched continuers within the same health system. The matched sample comprised 1,205 pairs with baseline balance (all standardized mean differences <0.1), but—like all nonrandomized designs—susceptibility to unmeasured confounding remains. Notably, the investigators complemented the primary matched analysis with a target-trial emulation sensitivity analysis, which produced concordant estimates, lending robustness to the observed associations. Accordingly, in our synthesis we treat the Geisinger findings as arising from a matched comparator design (rather than a true ‘control’ group) and weigh them with appropriate caution.

Upregulation of RAS contributes to the development of hypertension in CKD (Pugh et al., 2020). ACEi inhibits the conversion of angiotensin I (Ang I) to angiotensin II (Ang II), and ARB selectively prevents Ang II from binding to angiotensin II type I receptor (AT1R). Both drugs demonstrated a renoprotective effect due to their antihypertensive and antiproteinuric effects (Zheng et al., 2019). Proteinuria is strongly associated with the risk of CKD progression in both non-diabetic and diabetic patients. In non-diabetic CKD patients included the Ramipril Efficacy in Nephropathy (REIN) trial, urinary protein excretion was the only baseline variable that correlated with the rate of GFR decline and progression to ESKD (Ruggenenti et al., 1997). In according to two previous large RCTs on the effect of ARB in diabetic nephropathy, losartan and irbesatan, demonstrated ARB therapy was effective in protecting against the progression of nephropathy (Brenner et al., 2001). In a *post hoc* analysis of the REIN trial, the ramipril therapy was still beneficial for individuals with low eGFR, which decreased the rate of eGFR decline by 22% and the incidence of ESRD by 33% compared with the conventional group (non-ACEi treatment) (Ruggenenti et al., 2001). This *post hoc* analysis suggests that ACEi should not be withheld, even when eGFR approaches levels requiring replacement therapy.

Patients with CKD exhibit a pronounced risk for cardiovascular events. In a report, 50% of all patients with CKD stage 4–5 have cardiovascular disease (CVD) (Stevens et al., 2007). The traditional cardiovascular risk factors such as hypertension, dyslipidemia and insulin resistance are highly prevalent in patients with CKD (Roehm and Weiner, 2019; Zewinger et al., 2017). The hormones, enzymes, and cytokines in response to kidney injury or renal insufficiency lead to characteristic changes in the vasculature (Jankowski et al., 2021). ACEi reduce angiotensin II levels, thereby lowering blood pressure, but also prevent the breakdown of bradykinin to reduce both systemic and coronary resistance, thus providing additional cardioprotective effects (Roth et al., 2020). Although lack of individual trial or meta-analysis to prove ARB treatment on the incidence of cardiovascular evens, the European Society of Cardiology (ESC) guidelines still recommend ARBs in case of ACE inhibitor intolerance in patients at high cardiovascular risk (Knuuti et al., 2020). One network meta-analysis including 44 RCTs comprising 42,139 participants with non-dialysis CKD stage 3–5 found RAS blockade therapy increased the likelihood for hyperkalemia, hypotension, and cough. However it was still beneficial to protect kidney and cardiovascular functions (Zhang et al., 2020). Although the pool analysis in our study did not reach the significance (OR: 1.27, 95% CI: 0.93–1.73, P = 0.138), we could observe the trend of higher MACE risk when discontinuing ACEi/ARB in CKD stage 3–5 patients. This result might be limited by the limited number of included trials.

Hyperkalemia is a worrisome issue when continuing RASi in patients with impaired kidney function. Hsu et al. conducted a study enrolling 28,497 patients with serum creatinine levels more than 6 mg/dL. The result indicated that ACEi/ARB users (9.2%) had a higher risk of hyperkalemia-associated hospitalization than non-users (6.7%) (Hsu et al., 2014). In accordance with our results by enrolling three studies (Yang et al., 2023; Qiao et al., 2020; Hou et al., 2006), the pooled analysis showed lower incidence of hyperkalemia in discontinued group than the continued one (OR: 0.68, 95% CI: 0.60–0.77, P < 0.001).

Risk–benefit of RASi continuation. Discontinuation was associated with higher ESKD risk in our meta-analysis, whereas continuation increased hyperkalemia. In line with KDIGO 2024, we favor continuing ACEi/ARB with monitoring at 2–4 weeks and active hyperkalemia mitigation (dietary counseling, diuretics/sodium bicarbonate, potassium binders), reserving dose reduction/cessation for uncontrolled hyperkalemia. KDIGO’s algorithm explicitly sequences these steps and supports continuation even at eGFR <30 mL/min/1.73 m^2^, which frames the observed hyperkalemia as a manageable safety signal rather than a reason for routine discontinuation (Kidney Disease: Improving Global Outcomes, 2024).

To the best of our knowledge, this is the first systematic review and meta-analysis evaluating whether discontinuing RASi among CKD stage 3–5 patients is associated with poor outcome in terms of ESKD. Discontinuing RASi is associated with lower risk of hyperkalemia. Additionally, there is further evidence from the TSA, indicating that discontinued-RASi users has higher risk of ESKD than the continued-RASi users. However, this study had several limitations. First, Hou et al. conducted an RCT showing kidney benefits of benazepril in advanced CKD (Hou et al., 2006). However, because the primary outcome was a composite measure (doubling of serum creatinine, ESRD, or death), the individual outcomes for ESKD were not distinguishable, leading to the exclusion of this RCT from our meta-analysis. Second, our findings were predominantly extracted from observational studies. RCTs are anticipated to provide more solid strength. Third, the data extracted for the subgroup analyses from some enrolled studies lacked comprehensive information regarding the duration of CKD and the discontinuation of RASi, which could potentially bias our estimates. Forth, most included studies did not report RASi dose or exposure intensity in a standardized, comparable format, precluding a dose–response meta-analysis of hyperkalemia risk. Consequently, we cannot exclude residual confounding from clinical down-titration or temporary withholding of RASi in higher-risk patients, which may bias pooled estimates. Lastly, ACEi and ARB have different feedback in the RAAS; however we could not distinguish the two subgroups in our meta-analysis due to lack of data.

## Conclusion

Among patients with CKD stages 3–5, discontinuation of RASi therapy was associated with an increased risk of progression to ESKD. Continued use of RASi in this population raises concerns about hyperkalemia. Further large-scale randomized controlled trials are necessary to confirm these findings and provide more definitive evidence.

## Data Availability

The original contributions presented in the study are included in the article/Supplementary Material, further inquiries can be directed to the corresponding authors.

## Abbreviations

ACEi, angiotensin-converting enzyme inhibitors; ARB, angiotensin II receptor blockers; ADH, anti-diuretic hormone; Ang I, angiotensin I; Ang II, angiotensin II; AT1R, angiotensin II type I receptor; CI, confidence interval; CKD, chronic kidney disease; CVD, cardiovascular disease; eGFR, estimated glomerular filtration rate; ESKD, End-Stage Kidney Disease; ESC, European Society of Cardiology; GRADE, Grading of Recommendations, Assessment, Development and Evaluations; HR, hazard ratio; KDIGO, Kidney Disease Improving Global Outcomes; MACE, major adverse cardiovascular events; MeSH, Medical Subject Headings; NOS, Newcastle-Ottawa Scale; OR, odds ratio; PRISMA, Preferred Reporting Items for Systematic Reviews and Meta-Analyses; RAS, renin-angiotensin system; RASi, renin-angiotensin system inhibitors; RCTs, randomized controlled trials (RCTs); RIS, required information size; RoB 2.0, Version 2.0 of the Cochrane risk of bias; RR, relative risk; TSA, Trial sequential analysis.
