# Supplementary material for: Discontinuation versus continuation of renin–angiotensin system inhibitors in chronic kidney disease stage 3–5 patients: a systematic review and meta-analysis
Source: Front Pharmacol. 2025 Sep 26;16:1646969. doi: 10.3389/fphar.2025.1646969 (PMC12510950; doi:10.3389/fphar.2025.1646969)

**Supplementary Material**

**Discontinuation versus continuation of Renin–Angiotensin System Inhibitors in Chronic Kidney Disease Stage 3-5 patients: a Systematic** **Review and Meta-Analysis**

Authors: I-Wen Chen, Yi-Hsuan Lin, Vin-Cent Wu, Ming-Hsien Wu, Jui-Yi Chen

This supplementary material has been provided by the authors to give readers additional information about the work

1. PRISMA checklist.
2. Search equation via PubMed, Embase, and [Cochrane](http://www.cochranelibrary.com/) library.
3. Quality assessment of the included studies.

**Supplementary Table S1A.** Newcastle-Ottawa Scale Quality Assessment of included non-randomized controlled trials

**Supplementary Table S1B.** Risk of Bias 2.0 (RoB 2.0) of included randomized controlled trials

1. Subgroup analysis.

**Supplementary Figure S1.** The Forest plot for the odds ratio of (A) all-cause mortality and (B) ESKD comparing ACEi/ARB continuation versus discontinuation.

**Supplementary Figure S2.** The funnel plot showing the visual check for publication bias of the effect of discontinuing ACEi/ARB on the risk of (A) all-cause mortality and (B) ESKD by pooling the adjusted odds ratios.

**Supplementary Figure S3.** RCT versus non-RCT subgroup analysis of the pooled risk of (A) all-cause mortality (B) ESKD and (C) hyperkalemia between continuing and discontinuing ACEi/ARB groups

**Supplementary Figure S4.** CKD stage subgroup analysis of the pooled risk of (A) all-cause mortality (B) ESKD

1. Definitions of MACE and hyperkalemia in included studies.
2. Quality assessment the GRADE results.
3. Summary of contextual factor data.
4. PROSPERO protocol registration.
5. Abstract Graph.
6. **PRISMA checklist**

| **Section and Topic** | **Item** | **Checklist item** | **Location where item is reported** |
| --- | --- | --- | --- |
| **TITLE** | | |  |
| Title | 1 | Identify the report as a systematic review. |  |
| **ABSTRACT** | | |  |
| Abstract | 2 | See the PRISMA 2020 for Abstracts checklist. |  |
| **INTRODUCTION** | | |  |
| Rationale | 3 | Describe the rationale for the review in the context of existing knowledge. |  |
| Objectives | 4 | Provide an explicit statement of the objective(s) or question(s) the review addresses. |  |
| **METHODS** | | |  |
| Eligibility criteria | 5 | Specify the inclusion and exclusion criteria for the review and how studies were grouped for the syntheses. |  |
| Information sources | 6 | Specify all databases, registers, websites, organisations, reference lists and other sources searched or consulted to identify studies. Specify the date when each source was last searched or consulted. |  |
| Search strategy | 7 | Present the full search strategies for all databases, registers and websites, including any filters and limits used. |  |
| Selection process | 8 | Specify the methods used to decide whether a study met the inclusion criteria of the review, including how many reviewers screened each record and each report retrieved, whether they worked independently, and if applicable, details of automation tools used in the process. |  |
| Data collection process | 9 | Specify the methods used to collect data from reports, including how many reviewers collected data from each report, whether they worked independently, any processes for obtaining or confirming data from study investigators, and if applicable, details of automation tools used in the process. |  |
| Data items | 10a | List and define all outcomes for which data were sought. Specify whether all results that were compatible with each outcome domain in each study were sought (e.g. for all measures, time points, analyses), and if not, the methods used to decide which results to collect. |  |
|  | 10b | List and define all other variables for which data were sought (e.g. participant and intervention characteristics, funding sources). Describe any assumptions made about any missing or unclear information. |  |
| Study risk of bias assessment | 11 | Specify the methods used to assess risk of bias in the included studies, including details of the tool(s) used, how many reviewers assessed each study and whether they worked independently, and if applicable, details of automation tools used in the process. |  |
| Effect measures | 12 | Specify for each outcome the effect measure(s) (e.g. risk ratio, mean difference) used in the synthesis or presentation of results. |  |
| Synthesis methods | 13a | Describe the processes used to decide which studies were eligible for each synthesis (e.g. tabulating the study intervention characteristics and comparing against the planned groups for each synthesis (item #5)). |  |
|  | 13b | Describe any methods required to prepare the data for presentation or synthesis, such as handling of missing summary statistics, or data conversions. |  |
|  | 13c | Describe any methods used to tabulate or visually display results of individual studies and syntheses. |  |
|  | 13d | Describe any methods used to synthesize results and provide a rationale for the choice(s). If meta-analysis was performed, describe the model(s), method(s) to identify the presence and extent of statistical heterogeneity, and software package(s) used. |  |
|  | 13e | Describe any methods used to explore possible causes of heterogeneity among study results (e.g. subgroup analysis, meta-regression). |  |
|  | 13f | Describe any sensitivity analyses conducted to assess robustness of the synthesized results. |  |
| Reporting bias assessment | 14 | Describe any methods used to assess risk of bias due to missing results in a synthesis (arising from reporting biases). |  |
| Certainty assessment | 15 | Describe any methods used to assess certainty (or confidence) in the body of evidence for an outcome. |  |
| **RESULTS** | | |  |
| Study selection | 16a | Describe the results of the search and selection process, from the number of records identified in the search to the number of studies included in the review, ideally using a flow diagram. |  |
|  | 16b | Cite studies that might appear to meet the inclusion criteria, but which were excluded, and explain why they were excluded. |  |
| Study characteristics | 17 | Cite each included study and present its characteristics. |  |
| Risk of bias in studies | 18 | Present assessments of risk of bias for each included study. |  |
| Results of individual studies | 19 | For all outcomes, present, for each study: (a) summary statistics for each group (where appropriate) and (b) an effect estimate and its precision (e.g. confidence/credible interval), ideally using structured tables or plots. |  |
| Results of syntheses | 20a | For each synthesis, briefly summarise the characteristics and risk of bias among contributing studies. |  |
|  | 20b | Present results of all statistical syntheses conducted. If meta-analysis was done, present for each the summary estimate and its precision (e.g. confidence/credible interval) and measures of statistical heterogeneity. If comparing groups, describe the direction of the effect. |  |
|  | 20c | Present results of all investigations of possible causes of heterogeneity among study results. |  |
|  | 20d | Present results of all sensitivity analyses conducted to assess the robustness of the synthesized results. |  |
| Reporting biases | 21 | Present assessments of risk of bias due to missing results (arising from reporting biases) for each synthesis assessed. |  |
| Certainty of evidence | 22 | Present assessments of certainty (or confidence) in the body of evidence for each outcome assessed. |  |
| **DISCUSSION** | | |  |
| Discussion | 23a | Provide a general interpretation of the results in the context of other evidence. |  |
|  | 23b | Discuss any limitations of the evidence included in the review. |  |
|  | 23c | Discuss any limitations of the review processes used. |  |
|  | 23d | Discuss implications of the results for practice, policy, and future research. |  |
| **OTHER INFORMATION** | | |  |
| Registration and protocol | 24a | Provide registration information for the review, including register name and registration number, or state that the review was not registered. |  |
|  | 24b | Indicate where the review protocol can be accessed, or state that a protocol was not prepared. |  |
|  | 24c | Describe and explain any amendments to information provided at registration or in the protocol. |  |
| Support | 25 | Describe sources of financial or non-financial support for the review, and the role of the funders or sponsors in the review. |  |
| Competing interests | 26 | Declare any competing interests of review authors. |  |
| Availability of data, code and other materials | 27 | Report which of the following are publicly available and where they can be found: template data collection forms; data extracted from included studies; data used for all analyses; analytic code; any other materials used in the review. |  |

From: Page MJ, McKenzie JE, Bossuyt PM, Boutron I, Hoffmann TC, Mulrow CD, et al. The PRISMA 2020 statement: an updated guideline for reporting systematic reviews. BMJ 2021;372:n71. doi: 10.1136/bmj.n71

1. **Search equation via PubMed, Embase and** [**Cochrane**](http://www.cochranelibrary.com/) **library**

**Appendix.**

Search strategies for the different databases ran on May 30^th^, 2024.

**PubMed Search Query** (152)

(("advanced chronic kidney disease"[All Fields]) OR (chronic kidney disease)) OR ("end stage renal disease"[All Fields])) OR ("esrd"[All Fields])) AND (("renin angiotensin system inhibitors"[All Fields]) OR ("angiotensin converting enzyme inhibitor"[All Fields]) OR ("angiotensin receptor blocker"[All Fields]) OR ("acei"[All Fields]) OR ("arb"[All Fields])) AND (("discontinue"[All Fields]) OR (discontinued) OR ("continue"[All Fields]) OR ("continued"[All Fields]))

**EMbase** (210)

'advanced chronic kidney disease'/exp OR 'chronic kidney failure'/exp OR 'chronic kidney disease' OR 'chronic kidney disorder' OR 'chronic kidney failure' OR 'chronic kidney insufficiency' OR 'chronic nephropathy' OR 'chronic renal disease' OR 'chronic renal failure' OR 'chronic renal insufficiency' OR 'kidney chronic failure' OR 'kidney disease, chronic' OR 'kidney failure, chronic' OR 'kidney function, chronic disease' OR 'renal insufficiency, chronic' OR 'end stage renal disease'/exp OR 'esrd' OR 'end stage kidney disease' OR 'end stage kidney failure' OR 'end stage renal disease' OR 'end stage renal dysfunction' OR 'end stage renal failure' OR 'end stage renal impairment' OR 'end stage renal insufficiency' OR 'end-stage kidney disease' OR 'end-stage kidney failure' OR 'end-stage renal disease'

'renin angiotensin system inhibitor' OR 'angiotensin converting enzyme inhibitor' OR 'angiotensin receptor antagonist'/exp OR 'angiotensin ii receptor antagonist' OR 'angiotensin ii receptor antagonists' OR 'angiotensin ii receptor blocker' OR 'angiotensin ii receptor blockers' OR 'angiotensin ii receptor blocking agent' OR 'angiotensin ii receptor blocking agents' OR 'angiotensin receptor antagonist' OR 'angiotensin receptor antagonists' OR 'angiotensin receptor blocker' OR 'angiotensin receptor blockers' OR 'angiotensin receptor blocking agent' OR 'angiotensin receptor blocking agents' OR acei OR arb

discontinue OR continue

**Cocharne library** (158)

"advanced chronic renal failure" in Title Abstract Keyword OR "chronic kidney insufficiency" in Title Abstract Keyword AND "renin-angiotensin system" in Title Abstract Keyword OR "angiotensin receptor blocker" in Title Abstract Keyword AND "discontinuation" in Title Abstract Keyword

1. **Quality assessment of the included studies**

**Supplementary Table S1A. Newcastle-Ottawa Scale Quality Assessment of included non-randomized controlled trials**

|  | **Selection** | | | | **Comparability** | **Outcome** | | |  |
| --- | --- | --- | --- | --- | --- | --- | --- | --- | --- |
| First author / Year | **Representativeness of the exposed cohort** | **Selection of the non exposed cohort** | **Ascertainment of exposure** | **Demonstration that outcome of interest was not present at start of study** | **Comparability of cohorts on the basis of the design or analysis** | **Assessment of outcome** | **Was follow-up long enough for outcomes to occur** | **Adequacy of follow up of cohorts** | **Total** |
| Qiao et al. /2020 | * | * | * | - | * | * | * | * | 7 |
| Walther et al. /2021 | * | * | * | * | - | * | * | * | 7 |
| Fu EL et al. /2021 | * | * | * | * | * | * | * | * | 8 |
| Nakayama et al. /2022 | * | * | * | - | * | * | * | * | 7 |
| Yang A et al. /2022 | * | * | * | - | ** | * | * | * | 8 |
| Silva J. Leon et al. /2022 | * | * | * | - | * | * | * | * | 7 |

**Supplementary Table S1B. Risk of Bias 2.0 (RoB 2.0) of included randomized controlled trials**

**
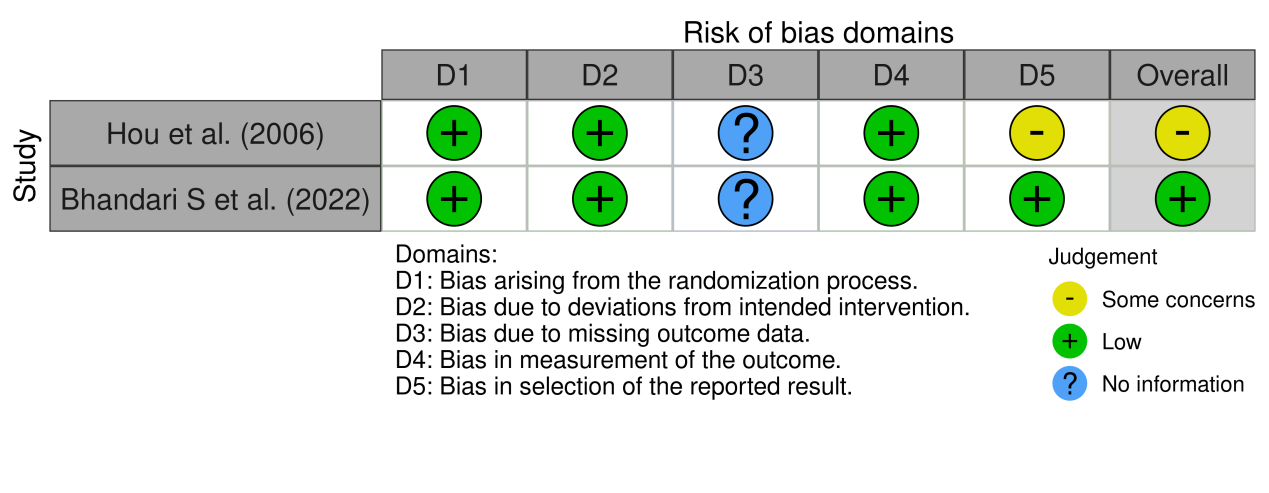
**

1. **Subgroup analysis**

**Supplementary Figure S1.** The Forest plot for the odds ratio of (A) all-cause mortality and (B) ESKD comparing ACEi/ARB continuation versus discontinuation.


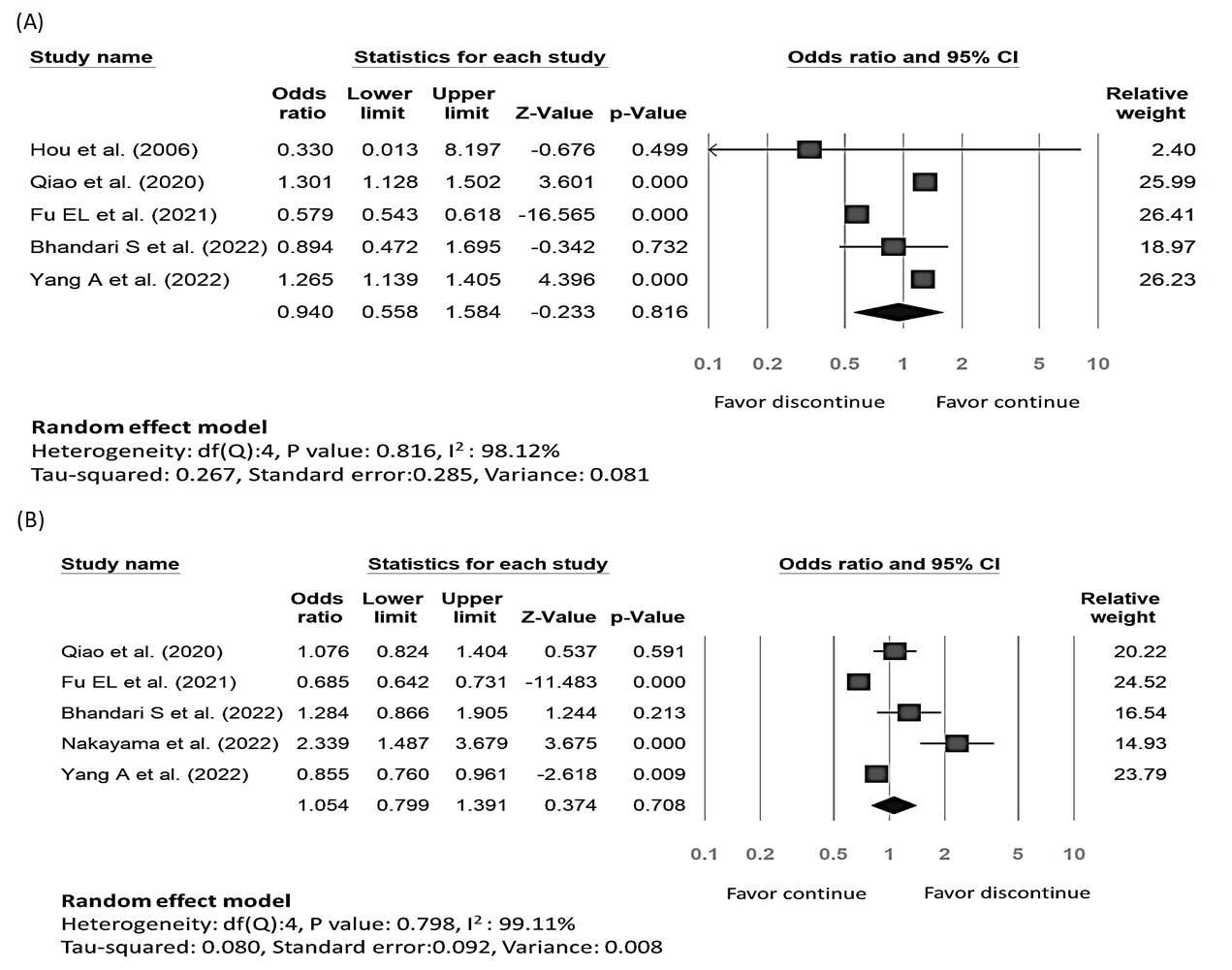


**Abbreviations.** ESKD, end-stage kidney disease; ACEi/ARB, angiotensin-converting enzyme inhibitor/angiotensin II receptor blocker

**Supplementary Figure S2.** The funnel plot showing the visual check for publication bias of the effect of discontinuing ACEi/ARB on the risk of (A) all-cause mortality and (B) ESKD by pooling the adjusted odds ratios.

**
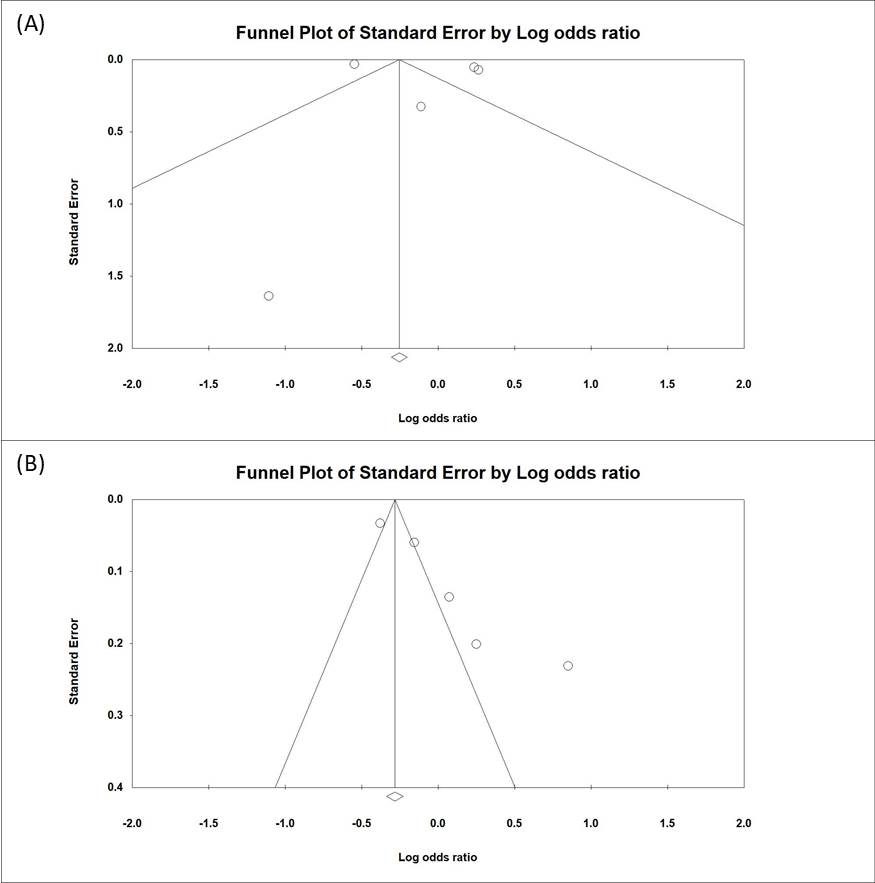
**

**Abbreviations.** ESKD, end-stage kidney disease; ACEi/ARB, angiotensin-converting enzyme inhibitor/angiotensin II receptor blocker

**Supplementary Figure S3.** RCT versus non-RCT subgroup analysis of the pooled risk of (A) all-cause mortality (B) ESKD and (C) hyperkalemia between continuing and discontinuing ACEi/ARB groups


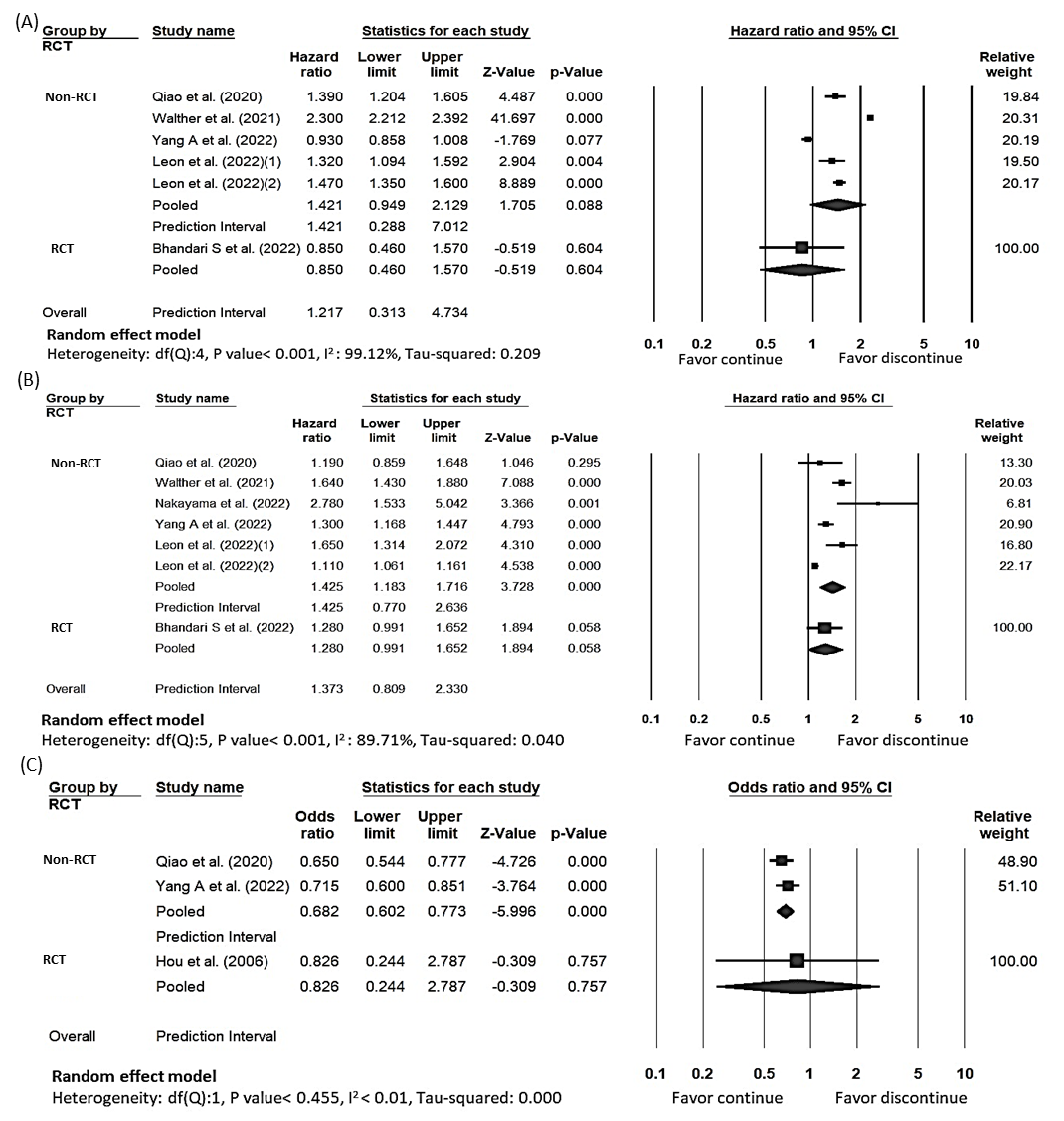


**Abbreviations.** RCT, Randomized controlled trials; ESKD, end-stage kidney disease; ACEi/ARB, angiotensin-converting enzyme inhibitor/angiotensin II receptor blocker

**Supplementary Figure S4.** CKD stage subgroup analysis of the pooled risk of (A) all-cause mortality (B) ESKD between continuing and discontinuing ACEi/ARB groups


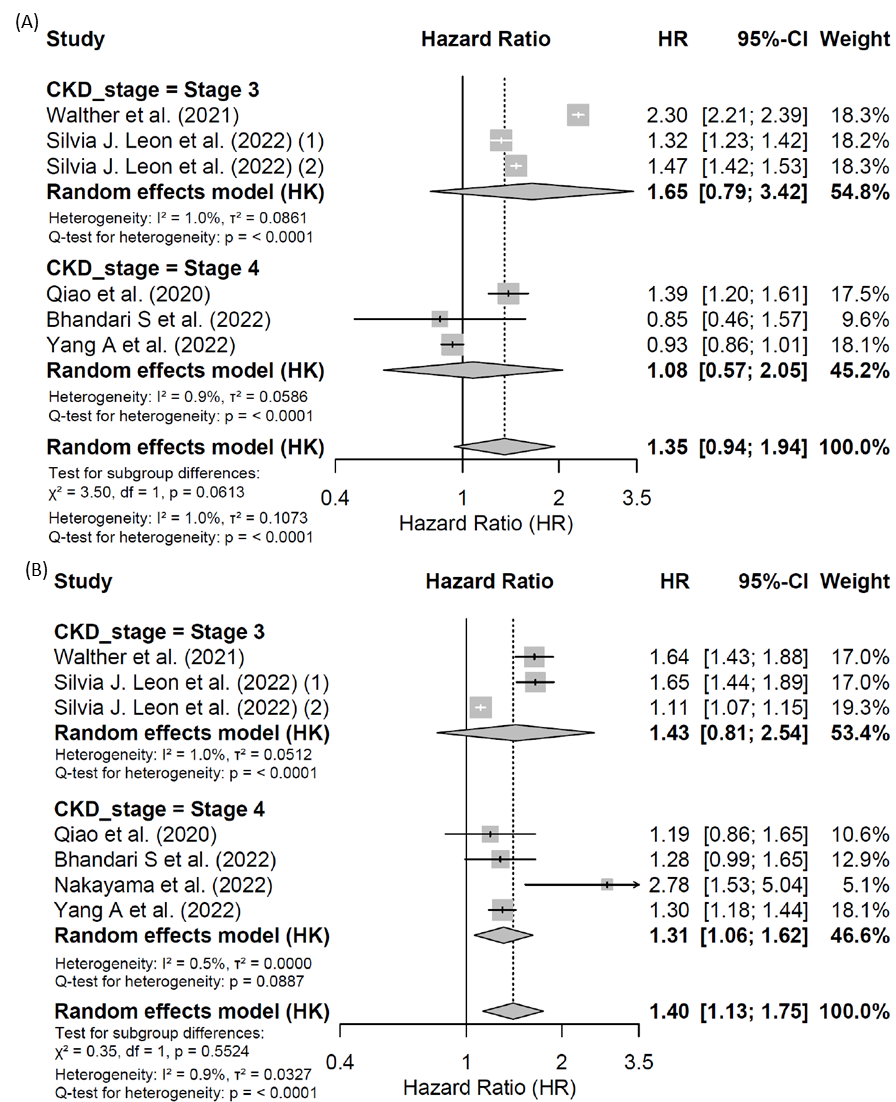


1. **Definitions of MACE and Hyperkalemia in Included Studies**

| **Study (year)** | **Design / Setting** | **MACE definition as stated in study** | **Hyperkalemia definition as stated in study** |
| --- | --- | --- | --- |
| **Hou et al., (2006)** | Randomized, placebo‑controlled trial | NR as a MACE composite; study tabulated “cardiovascular events” among adverse events rather than a prespecified MACE endpoint. | A serum potassium level ≥ 6 mmol/L |
| **Qiao et al., (2020)** | Target trial emulation in an observational cohort with propensity‑score matching | Composite of: death, myocardial infarction, percutaneous coronary intervention, or coronary artery bypass | First serum K> 5.5 mEq/L |
| **Fu et al., (2021)** | Target trial emulation using national registries | Composite of mortality, myocardial infarction, and cerebrovascular events | NR as an endpoint (study did not prespecify a hyperkalemia outcome). |
| **Walther et al., (2021)** | Observational cohort | NR (study focused on death and ESKD; no MACE composite reported) | NR as an endpoint (potassium discussed as a risk factor/context; not a prespecified hyperkalemia outcome) |
| **Yang et al., (2022)** | Population‑based cohort; complementary register‑based cohort for potassium outcomes | Composite of non‑fatal MI, non‑fatal stroke, and CV death | Plasma K ≥ 5.5 mmol/L (first event), excluding values during AKI hospitalizations in the register‑based cohort |
| **Bhandari et al., (2022)** | Multicenter randomized controlled trial (ACEi/ARB continuation vs withdrawal) | No prespecified MACE composite; trial reports counts of cardiovascular events (e.g., MI, stroke, arrhythmia, TIA, cardiogenic shock, etc.). | NR as an endpoint (hyperkalemia not formally defined as an outcome in the main report). |
| **Leon et al. (2022)** | Two‑province administrative‑data cohort among RASi users with de novo hyperkalemia | Study emphasized CV mortality and “fatal + non‑fatal cardiovascular events” (not a classic MACE label in the journal article) | NR as an endpoint (de novo hyperkalemia defined as a serum potassium level ≥ 5.5 mmol/L) |
| **Nakayama et al. (2022)** | Single-center retrospective study | NR (study focused on unplanned dialysis initiation; no MACE composite reported) | NR as an endpoint (hyperkalemia not formally defined as an outcome in the main report). |

**Abbreviations.** ARB, angiotensin receptor blocker; ACEi, angiotensin‑converting enzyme inhibitor; CV, cardiovascular; ESKD, end‑stage kidney disease; MI, myocardial infarction; MACE, major adverse cardiovascular events; NR, not reported; RASi, renin–angiotensin–aldosterone system inhibitor; TIA: transient ischemic attack

1. **Quality assessment of the GRADE results.**


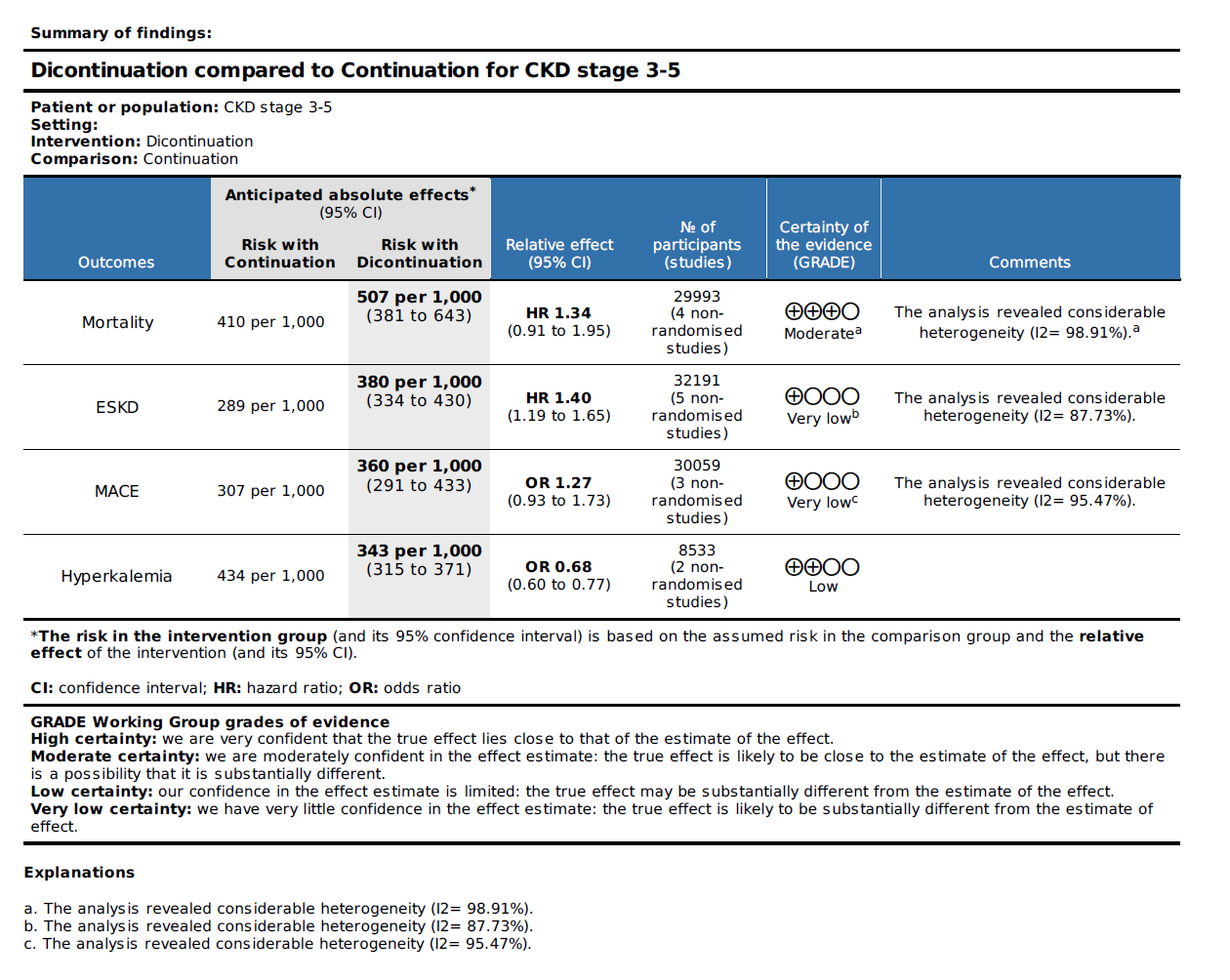


1. **Summary of contextual factor data**

In our meta-analysis, five retrospective studies, one prospective cohort study and two randomized control trials with 243,775 participants were included in this meta-analysis. In Hou et al.[20], 224 cases were included and 112 patients were administrated 20 mg of benazepril per day, who reached a 43 percent reduction in the risk of the primary end point of a doubling of the serum creatinine level, end-stage kidney disease (ESKD), or death (P = 0.005).

Qiao et al.[13] included 2,410 cases with chronic kidney disease (CKD) stage 4-5. Patients who discontinued angiotensin-converting enzyme inhibitor/ angiotensin II receptor blocker (ACEi/ARB) treatment had higher all-cause mortality (HR, 1.39; 95% CI, 1.20–1.61, P < 0.001) and major cardiovascular event (MACE) (HR, 1.29; 95% CI, 1.12–1.49, P < 0.001) but no increased the risk of ESKD events (HR, 1.19; 95% CI, 0.86–1.65, P = 0.295). Fu EL et al.[19] included 8,701 patients continuing and 1,553 discontinuing renin-angiotensin system inhibitors (RASi) with advanced stage CKD. The discontinuation group had higher MACE (HR, 1.62; 95% CI, 1.52–1.73, P < 0.001). Walther et al.[14] identified 141,252 people with non-dialysis CKD. Discontinuation of ACEi/ARB was associated with a higher risk of all-cause mortality (HR, 2.30; 95% CI, 2.21–2.39, P < 0.001) and ESKD (HR, 1.64; 95% CI, 1.43–1.88, P < 0.001). Bhandari S et al.[15] enrolled 411 patients with advanced stage CKD with median 3-year follow-up period. The discontinuation group had higher risk for developing of ESKD (HR, 1.28; 95% CI, 0.99–1.65, P = 0.058) but there was no significant for the risk of all-cause mortality between these two groups (HR, 0.85; 95% CI, 0.46–1.57, P = 0.604). Nakayama et al.[18] included 334 patients with CKD stage 5. Individuals with discontinuation of RAS inhibitors was significantly associated with a higher incidence of unplanned dialysis initiation (HR, 2.78; 95% CI, 1.53–5.04, P = 0.001). Silvia J. Leon et al.[17] reviewed two databases in Canada enrolling 7,200 patients in Manitoba and 71,290 in Ontario. Patients who discontinued ACEi/ARB treatment had higher risk of all-cause mortality (HR, 1.32; 95% CI, 1.09–1.59, P = 0.004 in Manitoba; HR, 1.47; 95% CI, 1.35–1.60, P < 0.001*-* in Ontario) and ESKD events (HR, 1.65; 95% CI, 1.31–2.07, P < 0.001 in Manitoba; HR, 1.11; 95% CI, 1.06–1.16, P < 0.001 in Ontario). Yang A et al.[16] included 10,400 patients discontinuing ACEi/ARB usage within 6 months after reaching estimated glomerular filtration rate (eGFR) < 30 ml/min/1.73 m^2^. Discontinuation of ACEi/ARB use was associated with higher risk of ESKD (HR = 1.30, 95% CI: 1.17–1.45, P < 0.001), and no increased risk of all-cause mortality (HR, 0.93, 95% CI: 0.86–1.01, P = 0.077) and MACE (HR, 0.95; 95% CI, 0.82–1.11, P = 0.537) compared to counterparts with continued use.

From most of the references in our manuscript, the usage of ACEi/ARB in CKD patients improved the mortality and delayed the progression of eGFR decline. The Kidney Disease: Improving Global Outcomes (KDIGO) Conference consensus suggested the first choice with RASi for patients with hypertension and CKD or increased albuminuria. However there is no robust conclusion regarding whether to continue RASi or not in patients with advanced stage CKD according to current evidence.

Of note, our data revealed continuation of RAS inhibitors in CKD stage 3-5 patients decrease the risk in developing ESKD. There was no significant benefit for all-cause mortality or cardiovascular events between discontinued/continued groups. However, hyperkalemia was observed in the continued group compared to discontinued group in CKD patients. Our meta-analysis incorporated a limited number of randomized controlled trials (RCTs), thus further research is anticipated to validate our findings.

1. **PROSPERO protocol registration**

1. * Review title.

Give the title of the review in English

The impacts of continuation or discontinuation of Renin–Angiotensin System Inhibitors in Chronic Kidney Disease Stages 3-5: A Meta-Analysis

2. Original language title.

For reviews in languages other than English, give the title in the original language. This will be displayed with the English language title.

The impacts of continuation or discontinuation of Renin–Angiotensin System Inhibitors in Chronic Kidney Disease Stages 3-5: A Meta-Analysis

3. * Anticipated or actual start date.

Give the date the systematic review started or is expected to start.

01/06/2023

4. * Anticipated completion date.

Give the date by which the review is expected to be completed.

31/01/2024

5. * Stage of review at time of this submission.

**This field uses answers to initial screening questions. It cannot be edited until after registration.**

Tick the boxes to show which review tasks have been started and which have been completed.

Update this field each time any amendments are made to a published record.

The review has not yet started: No

**Review stage Started Completed**

Preliminary searches Yes No

Piloting of the study selection process Yes No

Formal screening of search results against eligibility criteria Yes No

Data extraction No No

Risk of bias (quality) assessment No No

Data analysis No No

Provide any other relevant information about the stage of the review here.

6. * Named contact.

The named contact is the guarantor for the accuracy of the information in the register record. This may be　any member of the review team.

YI-Hsuan Lin

Email salutation (e.g. "Dr Smith" or "Joanne") for correspondence:

Dr Lin

7. * Named contact email.

Give the electronic email address of the named contact.

[yhl8304@gmail.com](mailto:yhl8304@gmail.com)

8. Named contact address

Give the full institutional/organisational postal address for the named contact.

No.5 Fusing St., Gueishan Dist., Taoyuan City 333, Taiwan

9. Named contact phone number.

Give the telephone number for the named contact, including international dialling code.

886-3-3281200 (ext. 8821)

10. * Organisational affiliation of the review.

Full title of the organisational affiliations for this review and website address if available. This field may be completed as 'None' if the review is not affiliated to any organisation.

Division of Endocrinology and Metabolism, Department of Internal Medicine, Chang Gung Memorial Hospital, Linkou branch, Taiwan

Organisation web address:

https://www.cgmh.org.tw/eng

11. * Review team members and their organisational affiliations.

Give the personal details and the organisational affiliations of each member of the review team. Affiliation refers to groups or organisations to which review team members belong. **NOTE: email and country now**

**MUST be entered for each person, unless you are amending a published record.**

Dr YI-Hsuan Lin. Division of Endocrinology and Metabolism, Department of Internal Medicine, Chang Gung Memorial Hospital, Linkou branch, Taoyuan

Dr I-Wen Chen. Division of Endocrinology and Metabolism, Department of Internal Medicine, Chang Gung Memorial Hospital, Linkou branch

Dr Ming-Hsien Wu. Division of Endocrinology and Metabolism, Department of Internal Medicine, Chang Gung Memorial Hospital, Linkou branch

12. * Funding sources/sponsors.

Details of the individuals, organizations, groups, companies or other legal entities who have funded or sponsored the review.

Nil

Grant number(s)

State the funder, grant or award number and the date of award

Nil

13. * Conflicts of interest.

List actual or perceived conflicts of interest (financial or academic).

None

14. Collaborators.

Give the name and affiliation of any individuals or organisations who are working on the review but who are　not listed as review team members. **NOTE: email and country must be completed for each person,**　**unless you are amending a published record.**

Professor VIN-CENT WU. National Taiwan University Hospital, Taipei

15. * Review question.

State the review question(s) clearly and precisely. It may be appropriate to break very broad questions down

into a series of related more specific questions. Questions may be framed or refined using PI(E)COS or

similar where relevant.

The impacts of continuation or discontinuation of RASi in chronic kidney disease stages 3-5 on all-cause mortality, end-stage kidney disease, cardiovascular events and hyperkalemia.

16. * Searches.

State the sources that will be searched (e.g. Medline). Give the search dates, and any restrictions (e.g. language or publication date). Do NOT enter the full search strategy (it may be provided as a link or attachment below.)

PubMed, MEDLINE, EMBASE, and the Cochrane Library were searched to locate relevant studies published

from the inception of these databases until 31th Jan., 2024.

17. URL to search strategy.

Upload a file with your search strategy, or an example of a search strategy for a specific database, (including

the keywords) in pdf or word format. In doing so you are consenting to the file being made publicly accessible. Or provide a URL or link to the strategy. Do NOT provide links to your search **results**.

Alternatively, upload your search strategy to CRD in pdf format. Please note that by doing so you are consenting to the file being made publicly accessible.

Do not make this file publicly available until the review is complete

18. * Condition or domain being studied.

Give a short description of the disease, condition or healthcare domain being studied in your systematic review.

Renin Angiotensin Aldosterone System inhibitor, chronic kidney disease, continue, discontinue

19. * Participants/population.

Specify the participants or populations being studied in the review. The preferred format includes details of　both inclusion and exclusion criteria.

The inclusion criteria were as follows: (a) individuals aged 18 years or older; (b) Chronic kidney disease stage 3-5 characterized by GFR 60 ml/min per 1.73 m2; (c) the administration of RASi, including either ACEi or ARB; (d) the presence of a control group to facilitate a comparison between the continuation or discontinuation of ACEi or ARB treatment; (e) the reporting of at least one outcome.

20. * Intervention(s), exposure(s).

Give full and clear descriptions or definitions of the interventions or the exposures to be reviewed. The

preferred format includes details of both inclusion and exclusion criteria.

The continue group are defined as those keep using Renin Angiotensin Aldosterone System inhibitor under

the status of chronic kidney disease stage 3-5

21. * Comparator(s)/control.

Where relevant, give details of the alternatives against which the intervention/exposure will be compared(e.g. another intervention or a non-exposed control group). The preferred format includes details of both　inclusion and exclusion criteria.

The discontinue group are defined as those don't use Renin Angiotensin Aldosterone System inhibitor under　the status of chronic kidney disease stage 3-5

22. * Types of study to be included.

Give details of the study designs (e.g. RCT) that are eligible for inclusion in the review. The preferred format includes both inclusion and exclusion criteria. If there are no restrictions on the types of study, this should be stated.

We will included randomized control study, prospective cohort study, retrospective cohort studies, and observational study to assess the outcomes of all-cause mortality, end-stage kidney disease, cardiovascular events and hyperkalemia.

23. Context.

Give summary details of the setting or other relevant characteristics, which help define the inclusion or

exclusion criteria.

24. * Main outcome(s).

Give the pre-specified main (most important) outcomes of the review, including details of how the outcome is defined and measured and when these measurement are made, if these are part of the review inclusion criteria.

All-cause mortality, end-stage kidney disease, cardiovascular events and hyperkalemia.

Measures of effect

Please specify the effect measure(s) for you main outcome(s) e.g. relative risks, odds ratios, risk difference, and/or 'number needed to treat.

25. * Additional outcome(s).

List the pre-specified additional outcomes of the review, with a similar level of detail to that required for main outcomes. Where there are no additional outcomes please state ‘None’ or ‘Not applicable’ as appropriate to the review

None

Measures of effect

Please specify the effect measure(s) for you additional outcome(s) e.g. relative risks, odds ratios, risk

difference, and/or 'number needed to treat.

Hazard ratio

26. * Data extraction (selection and coding).

Describe how studies will be selected for inclusion. State what data will be extracted or obtained. State how　this will be done and recorded.

The following data were extracted from the full-text articles: the first author name, year of publication, sample　size, study design, patient inclusion criteria, patient demographics, clinical outcome and adverse events.

27. * Risk of bias (quality) assessment.

State which characteristics of the studies will be assessed and/or any formal risk of bias/quality assessment　tools that will be used.

To assess the quality of the studies, we employed the Newcastle-Ottawa Scale (NOS) for cohort studies and　the Cochrane risk of bias tool for randomized controlled trials (RoB) in the case of RCTs

28. * Strategy for data synthesis.

Describe the methods you plan to use to synthesise data. This **must not be generic text** but should be **specific to your review** and describe how the proposed approach will be applied to your data. If metaanalysis is planned, describe the models to be used, methods to explore statistical heterogeneity, and　software package to be used.

For binary outcomes, a hazard ratio in conjunction with a 95% confidence interval (CI) would be conducted.

29. * Analysis of subgroups or subsets.

State any planned investigation of ‘subgroups’. Be clear and specific about which type of study or participant will be included in each group or covariate investigated. State the planned analytic approach.

Common variables include age, sex for meta-regression

30. * Type and method of review.

Select the type of review, review method and health area from the lists below.

Type of review

Cost effectiveness: No

Diagnostic: No

Epidemiologic: No

Individual patient data (IPD) meta-analysis: Yes

Intervention: Yes

Living systematic review: No

Meta-analysis: Yes

Methodology: No

Narrative synthesis: No

Network meta-analysis: No

Pre-clinical: No

Prevention: No

Prognostic: No

Prospective meta-analysis (PMA):No

Review of reviews：No

Service delivery：No

Synthesis of qualitative studies：No

Systematic review：Yes

Other：No

Health area of the review

Alcohol/substance misuse/abuse：No

Blood and immune system：No

Cancer：No

Cardiovascular：Yes

Care of the elderly：No

Child health：No

Complementary therapies：No

COVID-19：No

Crime and justice：No

Dental：No

Digestive system：No

Ear, nose and throat︰No

Education：No

Endocrine and metabolic disorders：No

Eye disorders：No

General interest：No

Genetics：No

Health inequalities/health equity：No

Infections and infestations：No

International development：No

Mental health and behavioural conditions：No

Musculoskeletal：No

Neurological：No

Nursing：No

Obstetrics and gynaecology：No

Oral health：No

Palliative care：No

Perioperative care：No

Physiotherapy：No

Pregnancy and childbirth：No

Public health (including social determinants of health)：No

Rehabilitation：No

Respiratory disorders：No

Service delivery：No

Skin disorders：No

Social care：No

Surgery：No

Tropical Medicine：No

Urological：No

Wounds, injuries and accidents：No

Violence and abuse：No

31. Language.

Select each language individually to add it to the list below, use the bin icon to remove any added in error.

English

There is not an English language summary

32. * Country.

Select the country in which the review is being carried out. For multi-national collaborations select all the countries involved.

Taiwan

33. Other registration details.

Name any other organisation where the systematic review title or protocol is registered (e.g. Campbell, or The Joanna Briggs Institute) together with any unique identification number assigned by them. If extracted data will be stored and made available through a repository such as the Systematic Review Data Repository(SRDR), details and a link should be included here. If none, leave blank.

34. Reference and/or URL for published protocol.

If the protocol for this review is published provide details (authors, title and journal details, preferably in Vancouver format)　Add web link to the published protocol.Or, upload your published protocol here in pdf format. Note that the upload will be publicly accessible.

No I do not make this file publicly available until the review is complete

Please note that the information required in the PROSPERO registration form must be completed in full even if access to a protocol is given.

35. Dissemination plans.

Do you intend to publish the review on completion?

No

Give brief details of plans for communicating review findings.?

36. Keywords.

Give words or phrases that best describe the review. Separate keywords with a semicolon or new line.

Keywords help PROSPERO users find your review (keywords do not appear in the public record but are included in searches). Be as specific and precise as possible. Avoid acronyms and abbreviations unless these are in wide use.

Renin-angiotensin system inhibitors; angiotensin-converting enzyme inhibitors; angiotensin II receptor blockers; chronic kidney disease; mortality; end-stage kidney disease; cardiovascular events; hyperkalemia

37. Details of any existing review of the same topic by the same authors.

If you are registering an update of an existing review give details of the earlier versions and include a full bibliographic reference, if available.

38. * Current review status.

Update review status when the review is completed and when it is published.New registrations must be ongoing so this field is not editable for initial submission.

Please provide anticipated publication date

Review_Ongoing

39. Any additional information.

Provide any other information relevant to the registration of this review.

40. Details of final report/publication(s) or preprints if available.

Leave empty until publication details are available OR you have a link to a preprint (NOTE: this field is not editable for initial submission). List authors, title and journal details preferably in Vancouver format.

1. **Abstract Graph**


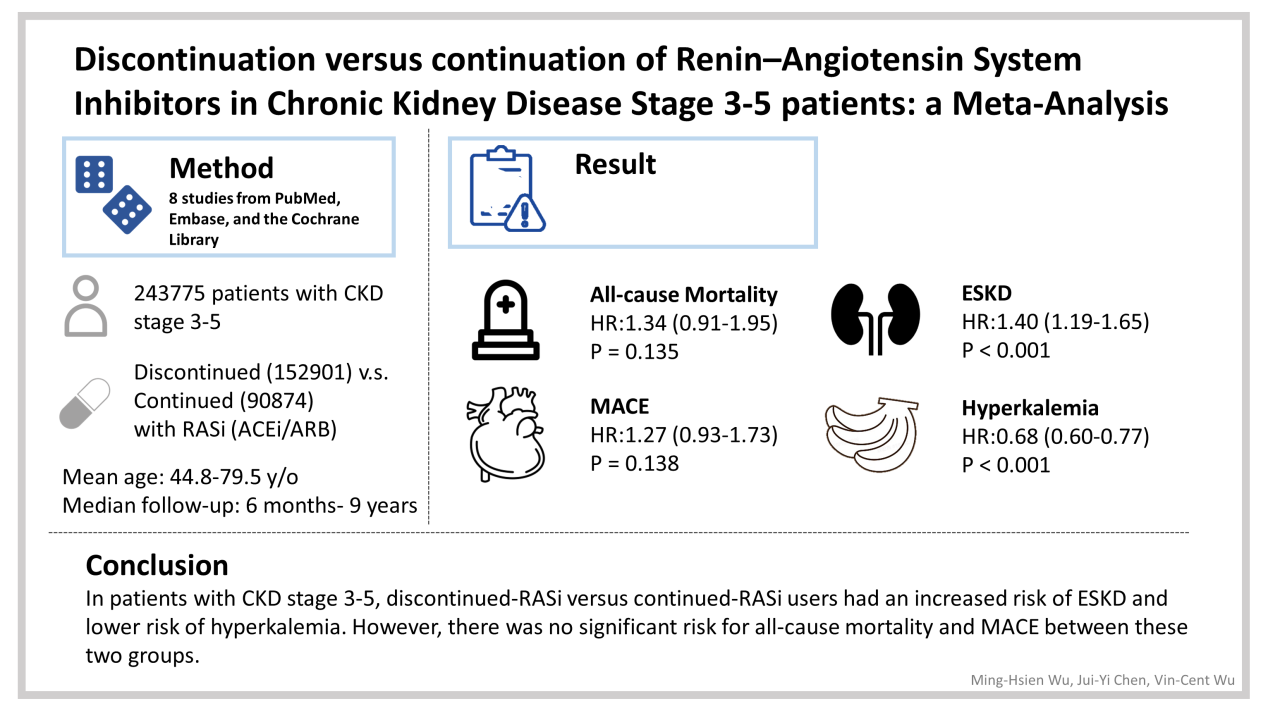

Supplement: Supplementary file 1 [file DataSheet1.docx]
